# Adenosine receptor 1 signal mediates the high salt diet induced tau pathology and behavioral abnormalities by suppression of Cers1

**DOI:** 10.1002/alz.085301

**Published:** 2025-01-03

**Authors:** Ling‐Qiang Zhu

**Affiliations:** ^1^ Department of Pathophysiology, School of Basic Medicine, Tongji Medical College, Huazhong University of Science and Technology, Wuhan China

## Abstract

**Background:**

Adenosine receptor 1 (A1R) is the predominant subtype of adenosine receptors, primarily distributed in memory‐associated brain regions such as the cortex, hippocampus, and cerebellum. It actively participates in plasticity‐regulated synaptic transmission and is crucial for functions related to sleep, arousal, cognition, learning, and memory. In a recent study, we reported that an elevation in A1R signaling mediates aberrant neuron‐glial crosstalk in Alzheimer’s disease. However, the role of A1R signaling in mediating tau pathology and behavioral abnormalities induced by a high salt diet, an independent risk factor for dementia and cognitive impairment, remains unclear.

**Method:**

We used western blot and qPCR to determine the expression of different protein and mRNAs. Morris water maze was used to examine learning and memory. Virus based shRNA delivery was used to silence the A1R and Cers1 expression

**Result:**

Here, we first reported that the Mef2c/miR‐133a/A1R signaling pathway is activated in the hippocampus of mice treated with a high salt diet (HSD). This activation is accompanied by tau pathology and disruption of neuron‐glial crosstalk. Application of KW3902, a specific A1R antagonist, or silencing of A1R, rescued tau pathology, neuron‐derived astrocytic activation, and synaptic dysfunction in HSD mice. Furthermore, we identified that the inhibition of Cers1, resulting from the deficiency of the cAMP‐guided pathway, led to both PP2a suppression and Lcn2 release, potentially underpinning the toxic effects of A1R activation in HSD mice. Artificial overexpression of Cers1 not only ameliorated tau pathology and astrocytic activation but also alleviated synaptic and memory impairments in HSD mice.

**Conclusion:**

Our study unveils a novel signaling pathway mediating high salt diet‐induced brain dysfunction and provides a new therapeutic target for related diseases.

